# Generation of human T cells directed against an agonist epitope of Brachyury, a transcription factor involved in human tumor cell epithelial to mesenchymal transition (EMT)

**DOI:** 10.1186/2051-1426-1-S1-P202

**Published:** 2013-11-07

**Authors:** Benjamin Boyerinas, Jo A Tucker, Diane J Poole, Caroline Jochéms, Claudia Palena, Jeffrey Schlom, Kwong Y Tsang

**Affiliations:** 1 Laboratory of Tumor Immunology and Biology, Center for Cancer Research, National Cancer Institute, National Institutes of Health, Bethesda, MD, USA

## Purpose

The T-box family transcription factor Brachyury is overexpressed in a variety of human carcinomas, including lung, breast, colon, ovarian and prostate. Brachyury has been shown to promote epithelial to mesenchymal transition (EMT) in tumor cells, a critical step in the path to metastasis. An HLA-A2 epitope of Brachyury has been shown to expand human T cells that are capable of lysing Brachyury-expressing tumor cells in an HLA-dependent manner. A phase I clinical trial is ongoing at the NCI using a recombinant yeast Brachyury vaccine. We have previously demonstrated that agonist epitopes of tumor-associated antigens are more effective than native epitopes at activating antigen-specific T cell responses. The current study sought to identify an agonist of the Brachyury HLA-A2 epitope in order to increase T cell activation and tumor lysis.

## Experimental design

A novel agonist epitope of Brachyury was generated by residue substitution of the native epitope. Characterization of this epitope as an agonist included; comparison of HLA-A2 binding affinity and stability, interferon-γ production by epitope-specific T cell lines, FACS analysis of activation markers, as well as Brachyury and HLA-A2 specific lysis of tumor cells. The presence of Brachyury-specific T cells in cancer patients that recognize the agonist peptide was determined by ELISPOT.

## Results

The agonist epitope was shown to bind HLA-A2 with higher avidity and stability than the native. PBMC from colon and ovarian cancer patients reacted to the agonist peptide in an ELISPOT assay. Tetramer staining revealed Brachyury agonist-specific T cells in the PBMC of a prostate cancer patient after in vitro stimulation with the agonist peptide. T cell lines generated from both the native and agonist epitopes produced higher levels of interferon-γ in response to stimulation with the agonist epitope, and they also expressed higher levels of intracellular Ki-67 and perforin. The agonist-specific T cell line lysed a variety of Brachyury-expressing tumor cells more efficiently.

## Conclusions

An agonist epitope for Brachyury has been identified that, as compared to the native epitope, increased T cell activation and cytotoxic activity against tumor cells expressing native Brachyury. A T cell line generated with the agonist peptide displayed increased activation (Ki67, perforin) and cytotoxic activity upon subsequent stimulation with the peptide. This study supports the use of the Brachyury agonist epitope as a cancer vaccine strategy.

